# Beyond the Outbreak of COVID-19: Factors Affecting Burnout in Nurses in Iran

**DOI:** 10.5334/aogh.3190

**Published:** 2021-06-24

**Authors:** Sajad Zare, Reza Kazemi, Abolhassan Izadi, Andrew Smith

**Affiliations:** 1 Department of Occupational Health, School of Health, Kerman University of Medical Sciences, Kerman, Iran; 2 Department of Ergonomics, School of Health, Shiraz University of Medical Sciences, Shiraz, Iran; 3 Centre for Occupational and Health Psychology, School of Psychology, Cardiff University, Cardiff, UK

## Abstract

**Background:**

Nurses working in treating patients with COVID-19 are exposed to various stressors, such as fear of COVID-19, stress, and high workload, leading to burnout.

**Objectives:**

This study aimed to identify the level of burnout and its predictors in nurses working in hospitals for COVID-19 patients.

**Methods:**

Participants in this study were nurses working in 11 hospitals for COVID-19 patients in the Fars province of Iran. The Maslach burnout and the UK Health and Safety stress questionnaires were used to assess burnout and stress, respectively. Analysis, using multiple regression in the SPSS21 software, aimed to identify the factors affecting burnout.

**Findings:**

The mean level of burnout in the nurses at the COVID-19 hospitals was 57 out of 120, and burnout was affected by workload (β = 0.69, p < 0.001), job stress (β = 0.25, p < 0.001) and inadequate hospital resources for the prevention of COVID-19 (β = –0.16, p < 0.001). These three variables explained 87% of the variance in burnout.

**Conclusions:**

The burnout of nurses directly exposed to COVID-19 patients is more than nurses in other wards, and workload is the most significant cause of burnout in them. Therefore, necessary measures such as hiring more nurses, reducing working hours and increasing rest periods are necessary to reduce workload. In addition, the job stress of these nurses should be managed and controlled, and the hospital resources needed to prevent this disease should be provided.

## Introduction

Burnout is the outcome of long-term exposure to job demands and stress [1]. It is a physical, mental, and emotional syndrome that includes pessimism about oneself and the job, causes a severe reduction in desire to do the job, and can lead to increased turnover [2]. According to a review of studies in the past 25 years, 26% of nurses suffer from burnout [3]. This syndrome is strongly associated with working conditions in the hospital [4]. In general, burnout is due to personal factors and factors relating to the workplace [5]. Past studies also indicated that personal factors affecting burnout included demographic characteristics, gender, age, religion, education level, having children, living with family, personality, job stress, coping strategies, and job attitudes [2,3,5]. Job factors affecting burnout include exposure to traumatic events, workload, type of employment, working hours, support of officials, wages, social support, and lack of financial resources [5].

The outbreak of an unknown disease called COVID-19 in Wuhan, China and its spread worldwide has led to the most significant pandemic of the century [6]. Iran was one of the first countries where the disease spread; it infected about 600,000 people and killed more than 30,000 people, including about 300 medical personnel, in the period before the present study [7]. The high prevalence rate in Iran has increased the anxiety and risk of health workers and their workload. Nurses working in hospitals for patients with coronavirus are among the highest risk groups for the disease, which can cause excessive stress [8]. Furthermore, they are responsible for a large amount of the care and treatment of COVID-19 patients, which can lead to burnout and turnover [9]. A few studies have shown that nurses and frontline staff dealing with COVID-19 are more prone to burnout than other nurses and health care workers [10,11]. In addition to these studies, the risks of SARS mortality, job stress, and their impact on social and family relationships have been considered as factors affecting nurses’ turnover during the years of the SARS outbreak [12]. During the rapid spread of the H5N1 virus in Taiwan, nurses’ fear of getting sick greatly influenced their willingness to care for patients [13]. Guadalupe Manzano García and her colleague showed that the threat of COVID-19 affects nurses’ burnout through mediators such as workload, human and materials resources, and social support in the workplace [10].

An investigation of the factors affecting burnout in emergency department nurses of eight hospitals in South Korea during the outbreak of MERS indicated that job stress, insufficient hospital resources in disease prevention, and lower support of family and friends were crucial factors in nurses’ burnout [2]. Since the disease has become a pandemic, the health care managers should also maintain the physical and mental health of medical staff, especially nurses, and try to improve the performance of medical staff and efforts to reduce casualties and increase the number of recovered patients. Identifying the factors affecting burnout is very important for its management and prevention. The effect of the COVID-19 pandemic on nurses is largely unknown, and therefore, the present study aimed to investigate the levels of burnout and its determinants in hospitals treating COVID-19 patients. The research also investigated possible solutions for burnout prevention.

## Methods

### Study design

The present cross-sectional study was conducted to evaluate the determinants of burnout in nurses working on the front lines of fight with COVID-19. Data were collected from the beginning of September 2020 to October 20, 2020.

### Setting and sample

The sample included nurses from 11 hospitals treating COVID-19 patients in a southern province of Iran, Fars. All participants signed the consent forms before responding to the questionnaires. The questionnaires were given to the participants via WhatsApp and Telegram virtual networks and were completed online. A sample size calculation using G*Power 3.1 suggested an N = 180, and data were obtained from 220 nurses (20 from each hospital). Two hundred ten nurses (about 95%) completed the questionnaires, and 208 were included in the final analysis after excluding the uncompleted questionnaires.

## Measurements

### COVID-19-related burnout

The Persian version of the Maslach Burnout Inventory (MBI) was used to evaluate burnout. To limit and link the study to COVID-19, the term “caused by COVID-19” was added to each item. MBI consists of 22 items that generally constitute the burnout assessment index and uses a 7-point Likert scale (0: never occurs to me, 6: it occurs to me every day) for the evaluation.

Based on the total score, burnout was sub-divided into no burnout (below 50), mild burnout (50–75), moderate burnout (75–100), and severe burnout (above 100). Cronbach’s alpha of the questionnaire was 0.78 in previous studies [14].

### COVID-19-related job stress

The Persian version of the 10-item HSE questionnaire was used to evaluate job stress. Like the Maslach inventory, the term “caused by COVID-19” was added to each question to limit the study to COVID-19. A 5-point Likert scale, including 1 (strongly disagree) to 5 (strongly agree), was used for answering the questionnaire. Cronbach’s alpha of the questionnaire was 0.74 in previous studies [15].

### Workload due to COVID-19

In the present study, the workload evaluation was based on previous studies using the term “My workload has increased due to COVID-19”, which was answered using a 10-point visual rating index. A higher response score meant a higher workload.

### Hospital resources for the prevention of COVID-19

A questionnaire developed by Ji Soo Kim et al. was used to evaluate the resources and programs of hospitals used to prevent COVID-19. To this end, the questionnaire was first translated into Persian and then validated [2]. Cronbach’s alpha of the questionnaire was 0.78, and its content validity was 0.95.

The questionnaire consisted of the following three items: “My hospital is equipped with sufficient facilities for preventing the spread of COVID-19”, “My hospital applies the best infection control guideline for preventing the spread of COVID-19”, and “My hospital discusses how to prevent COVID-19 regularly”. The questionnaire was answered using a 4-point Likert scale ranging from 1 (strongly disagree) to 4 (strongly agree).

### Support from family and friends

We used a questionnaire by Ji Soo Kim et al. to assess the support of family and friends from the participants [2]. The questionnaire was translated into Persian and validated using a Cronbach’s alpha of 0.75 and a content validity of 0.83. This questionnaire consisted of the following four items: “My friends will avoid me if they find that I have cared for COVID-19 patients”,” My friends will support me caring for COVID-19 patients”,” My family will avoid me if they find that I have cared for COVID-19 patients”, and “My family will support me caring for COVID-19 patients”. In this index, each question was answered using a 4-point Likert scale (1: strongly disagree and 4: strongly disagree with the support from friends and family).

## Data analysis

SPSS21 was used to analyze the data. First, the data normality was examined using the Kolmogorov-Smirnov test. The frequency, mean, percentage, and standard deviation indices for measuring demographic characteristics, job stress, workload, and support from family and friends, and hospital resources to prevent COVID-19 were then calculated. An analysis of variance (ANOVA) was used to assess differences in burnout based on personal characteristics. The associations between variables were then examined using the Pearson’s correlation test, followed by multiple regression.

## Results

In the present study, the participants’ mean age was 25.7 (5.2) years, and 55.5% were male. Of the participants, 80% were married, and 84.5% had at least a bachelor’s degree. Their clinical experience was about 12 years. Fifty-eight percent worked three shifts (morning, evening, and night), and the rest were day workers. Of the participants, 54% of them had a history of direct exposure to COVID-19 patients. There was no difference between the two groups, those who had been exposed and those who had not, in baseline demographic characteristics (***Table 1***).

**Table 1:** Comparison of demographic characteristics and main variables between the two groups.


VARIABLES		EXPERIENCE IN CARING FOR COVID-19 INFECTED		p

		YES(N = 112)	NO(N = 96)	

**age**		25.4	25.9	0.23

**Gender**	Men	64	52	0.70

	women	48	44	

**Marital statues**	Single	29	19	0.08

	married	83	77	

**Child**	No	51	45	0.078

	yes	61	51	

**Education level**	BS	88	80	0.09

	MS	24	16	

**Work schedule**	Day shift	42	46	0.06

	Rotating 3-shift	70	50	

**Exercise**	No	60	54	0.237

	yes	52	42	

**stress**		39(8)	33(6)	0.04

**Burnout**		57(23)	50.5(20)	0.03


The participants’ mean burnout score was 54 out of 132. Furthermore, the job stress score was obtained 36 out of 50 and the workload score of 5.4 out of 6. Family support score was 3 out of 4, and friend support score was 3 out of 4.

**Figure 1:**
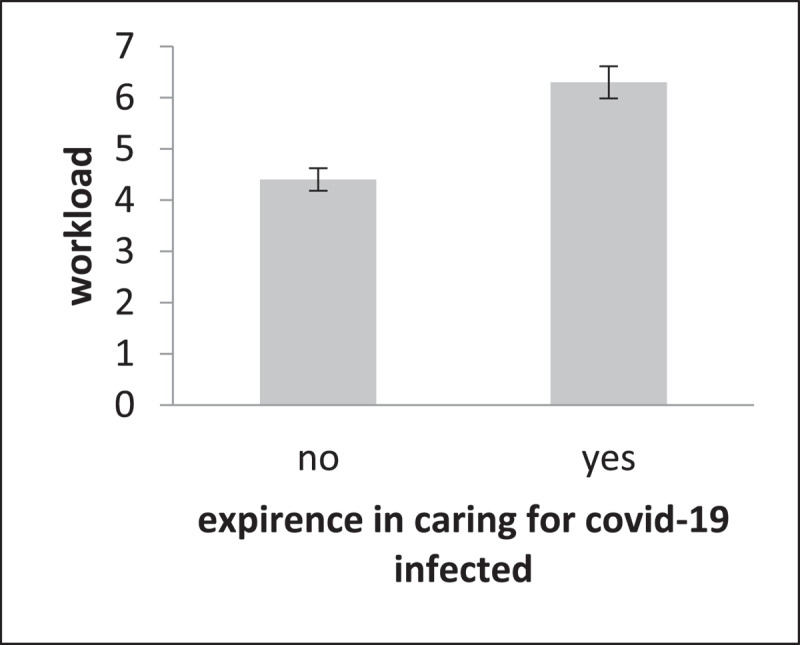
Comparison of workload between two groups of direct exposure to COVID-19 and indirect exposure to COVID-19.

COVID-19 burnout and stress were significantly higher in people who had direct exposure to COVID-19 patients (p < 0.05; see ***Table 1***). Also, the workload was significantly higher in nurses who had experience in caring for COVID-19 patients (see ***Figure 1***).

The level of burnout was higher in single nurses (p = 0.01), nurses working the three-shift system (p = 0.01), and nurses who feared infection (p = 0.001; see ***Table 2***).

**Table 2:** General Characteristics and Differences in COVID-19-related Burnout (N = 208).


VARIABLES	CHARACTERISTICS	N (%)	BURNOUT MEAN ± SD	T OR F(P)

Gender	Men	116	55(21)	2.21 (0.15)

	women	92	60(23)	

Marital status	Single	48	64(24)	0.44 (0.01)

	married	160	55(21)	

Child	No	56	61(25)	1.60 (0.03)

	yes	92	52(21)	

Education level	BS	168	58(23)	2.40 (0.1)

	MS	40	52(20)	

fear	yes	144	60(22)	3.10 (0.01)

	no	63	51.5(21)	

Work schedule	Day shift	88	45)17)	5.7(0.001)

	Rotating 3-shift	120	66(22)	

exercise	no	136	54(24)	16(0.07)

	yes	64	60(17)	


### Factors influencing COVID-19-related burnout

Multiple regression analysis indicated that the workload was the most important cause of burnout (β = 0.69, p < 0.001). Job stress and the scarcity or inadequacy of hospital resources for preventing COVID-19 were also identified as determinants of burnout caused by COVID-19. These three factors accounted for 87% variance in the incidence of burnout (see ***Table 3***).

**Table 3:** Linear Regression Analysis for Influencing COVID-19 related Burnout (N = 208).


	B	SE	β	T	P	CI		F (P)	R^2^	ADJUSTED R^2^

						LOWER	UPPER			

Constant	–18.625	5.818		–3.201	.002	–30.099	–7.151	149(0.001)	0.872	0.866

shift	3.281	1.649	.071	1.989	.048	.028	6.533			

workload	7.205	0.516	.693	13.960	.000	6.187	8.223			

Experience in caring for COVID-19 infected	1.848	1.644	.040	1.124	.262	–1.394	5.089			

Hospital resources for prevention of COVID-19	5.270	0.929	–.163	–5.675	.000	–3.439	–7.101			

Support from family & friends	–.932	1.205	–.022	–.773	.440	–3.308	1.445			

stress	.364	0.067	.251	5.436	.000	.232	.496			

Marital status	.313	2.126	.006	.147	.883	–3.879	4.504			

Fear of COVID-19 infection	1.404	1.470	.028	.955	.341	–1.495	4.302			

child	1.256	1.225	.041	1.026	.306	–1.159	3.671			


a. Dependent Variable: burnout.

## Discussion

The present study aimed to determine the level of burnout and its determinants in nurses who cared for the COVID-19 patients. The study investigated factors identified in previous studies as predictors of nurses’ burnout during the outbreak of SARS and MERS [13]. Stress, social and familial support, and lack of appropriate preventive facilities at hospitals were factors that were known as determinants of burnout during the outbreak of infectious lung diseases similar to COVID-19. Workload was another major factor identified as a cause of burnout in the medical staff in previous studies. Iran is the second country in Asia with the highest prevalence of coronavirus, with over 300,000 people infected and 30,000 dead from the virus before the present study [7]. Therefore, nurses working in hospitals for COVID-19 patients are more likely to suffer from burnout, and it is essential to know its determinants.

The burnout score of nurses, who experienced direct exposure to COVID-19 patients, was 57 out of 132, and the burnout score of 54% of nurses was more than 50. Based on the MBI, A score of 50 to 75 is mild burnout. Therefore, nurses suffered from mild burnout in the present study [14]. However, since only six months passed from the outbreak of COVID-19 until the present study, the rate of burnout was significant and might increase if not prevented. Previous studies on nurses indicate that the prevalence of burnout is from 19% to 25%; hence, the present results indicated that the prevalence of burnout in nurses, who were at the forefront of COVID-19, was much higher than the mean score of previous studies, and thus there is a need for immediate significant preventative measures [3,16].

The univariate analysis indicated that being married and having children affected burnout, but they were not significant determinants of burnout in multiple analyses. Previous studies have shown that demographic factors such as age, gender, having children, and marriage are determinants of burnout, which contradicts the present study results. The present results indicated that having children and marriage indirectly, for instance, through stress and workload, could affect burnout [5,17].

Previous studies report that workload is a determinant of burnout [5]. In the present study, the workload was the most important and significant cause of burnout, and there was a direct relationship between workload and burnout, with increasing workload leading to enhanced burnout. The nurses’ workload has increased sharply due to the increase in the presence of COVID-19 patients. Furthermore, excessive work reduced and depleted the individuals’ energy and occupied the nurses’ rest time and energy recovery, leading to burnout; hence, the hospital managers and decision-makers need to reduce workload as it is the leading cause of burnout [18].

Work-related stress is another important determinant of burnout that was reported as a significant factor in previous studies. Studies on the impact of MERS on nurses’ burnout showed that stress was the strongest predictor [14]. In the present study, there was a significant relationship between stress and burnout, with increasing job stress also enhancing burnout. According to the multivariate analysis, job stress was a significant determinant of burnout. The research results were consistent with a study by Ji Soo Kim et al., [2] who found that stress was the primary determinant of burnout during the outbreak of MERS-COV. The constant referral of patients with COVID-19 and the spread of infection to nurses were reasons for the higher stress in nurses.

In addition to workload and stress, the most significant causes of burnout in nurses in the present study were the inadequate resources and measures in hospitals used to prevent COVID-19. Nurses’ burnout decreased with increases in the preventive measures and the provision of preventative resources. The results were consistent with a systematic review of studies that reported that preventative resources in hospitals affected burnout. They were also consistent with a study by Ji Soo Kim et al., who reported that the preventative resources of MERS affected nurses’ burnout. The findings indicated that it is necessary to increase the staff safety against COVID-19 by providing necessary facilities and improving safety to prevent burnout in the nurses [2].

## Limitations

The first limitation of the present study was that it included only one country; hence, there is a need for further studies in other countries to examine the risk factors. The present study did not examine factors such as personality, job attitude, coping strategies, and salary shown in previous studies to be determinants of burnout. The increase in burnout due to occupational and environmental factors in the nursing profession has been proven, so part of the current burnout may have existed pre-COVID. These factors were not directly addressed in the present study, and this is another limitation. However, comparisons with levels of burnout reported in the literature before COVID-19 show higher levels in the present sample. A combination of qualitative and cross-sectional methods should also be used in future research to understand the topic better.

## Conclusion

In addition to disease prevention, managers of hospitals and treatment centers in any country must pay attention to the mental health of the nurses who are at the forefront of COVID-19. Although burnout is a common disorder in nurses, the present study indicated that nurses in COVID-19 wards were more prone to burnout, and their burnout was higher than nurses in non-Coronavirus wards and other previous studies. The workload was the leading cause of burnout in nurses in the present study. Stress and weakness of hospital resources were other factors affecting burnout. The three factors accounted for 86.6% of the variance of burnout caused by COVID-19. The research results provided important information about the development of prevention strategies for burnout caused by COVID-19 in nurses and treatment staff. Therefore, reducing the nurses’ workload and stress and developing the hospital resources for preventing COVID-19 should be priorities in managers’ plans to prevent burnout in nurses.
